# Comparative Ultrasonographic Imaging of Spleen and Liver in Healthy Crossbred Cows

**DOI:** 10.5402/2011/419591

**Published:** 2012-01-11

**Authors:** Sheikh Imran, S. P. Tyagi, Amit Kumar, Adarsh Kumar, Shivali Sharma

**Affiliations:** Department of Veterinary Surgery and Radiology, College of Veterinary & Animal Sciences, Chaudhary Sarwan Kumar Himachal Pradesh Krishi Vishvavidyalaya (CSKHPKV), Himachal Pradesh, Palampur 176 062, India

## Abstract

The present study was undertaken to conduct a comparative ultrasonographic study of the normal bovine spleen and liver. This study was carried out in two phases using 3.5 MHz curvilinear transducer. In first phase of water bath study, bovine spleens and livers were obtained from 3 healthy cadavers and subjected to repeated ultrasonography to study the echotexture. The splenic parenchyma was isoechogenic with greater echogenicity as compared to the liver. Water bath study provided a good learning experience for the comparative analysis of echotexture of the spleen and liver. In second phase, 10 healthy Jersey/Red Sindhi adult nonpregnant cows were subjected to detailed abdominal ultrasonography a number of times to develop the baseline topographical data of the spleen and liver. The dorsal end of the spleen in the cranial intercostal spaces could be clearly seen at the peak of inspiration when the lungs retracted to uncover the parietal surface of the spleen. The ventral end of the spleen, portion of the left lung, and the reticular wall could be scanned in the left 7th to 6th ICS in all the cows, and in the left 5th ICS in 5 cows. The liver was imaged from just behind the 12th to 6th ICS in all the cows easily. The gallbladder was imaged as a tear drop or pear shaped anechogenic structure with a hyperechogenic wall. The location of the gallbladder varied from the 12th to 9th ICS. It was most consistently observed in the 11th ICS (in 7 cows) at a mean distance of 46.3 cm from the dorsal midline. In 3 cows, the gallbladder was also seen ventral to the costal arch. The caudal vena cava was imaged as a triangular anechogenic structure on the dorsal border of the liver in the last 2 intercostal spaces in all the cows. The portal vein was imaged characteristically as a star shaped anechogenic structure with a hyperechogenic wall, ventral to the caudal vena cava, in the last 2 intercostal spaces in all the cows and in the 10th ICS in only 8 cows. It was concluded that a systematic ultrasonography formed a basis for a reliable noninvasive determination of positions and sizes of the normal spleen and liver and their vessels in the bovines.

## 1. Introduction

 The spleen is not easily accessible for clinical examination by palpation or percussion, because of its topographic location under costal part of the abdominal wall. It can neither be examined by rectal palpation nor radiographed, thus making the diagnosis of splenic conditions very difficult. Spleen is often affected by other disorders of the gastrointestinal tract such as traumatic reticuloperitonitis/pericarditis and rarely by tumors such as lymphoma [1]. Exploratory laparotomy confirms the diagnosis of splenic conditions, although its invasiveness may not be suitable in already compromised patients. Veterinary laboratory diagnosis is also a nonspecific indicator of the splenic diseases. Similarly, clinical diagnosis of the liver diseases is difficult in cattle, since even severe diseases may not always be accompanied by specific signs like jaundice. The common laboratory tests for liver function also usually fail to reveal a disorder as long as one-third of the liver parenchyma is still functioning, and there is no impairment of bile flow [2]. Diagnostic ultrasonography, however, may enable the clinician to get an accurate assessment of at least part of the spleen/liver. Therefore, a normal ultrasonographic baseline data will be valuable in the cows with suspected splenic/liver disease. Ultrasonographic studies describing the normal topographic and textural characteristics of the spleen and liver are rare [1, 3]. Comparative hepatic/splenic water bath studies and breed-based variations in the ultrasonographic topography of spleen and liver in cattle have not been reported until now. Therefore, the present study was undertaken to conduct a comparative ultrasonographic study of normal spleen and liver in the healthy crossbred cows.

## 2. Materials and Methods 

### 2.1. Water Bath Study

Whole spleens and livers were collected from 3 apparently healthy adult bovine cadavers that were euthanized at the teaching veterinary clinical complex, CSKHPKV, Palampur, for disorders unrelated to thoracoabdominal disease. The apparently normal organs were collected within 3 hours after death. The organs were separately immersed in a water bath and examined to determine their respective ultrasonographic appearances. A 3.5 MHz curvilinear transducer secured in a water-tight gel-laden polythene covering was used for imaging.

### 2.2. Ultrasonography of the Spleen and Liver in Healthy Cows

Examinations were performed on 10 non-pregnant, clinically healthy Jersey/Red Sindhi crossbred cows. The cows were considered to be clinically healthy based on the results of routine physical examination and a complete blood count; however, none of the cows could be slaughtered for gross examination of spleen and liver due to imposition of ban on cow slaughter as per the directive principles of state policy enumerated in part IV of the Indian Constitution. The cows were between 4–12 years old and weighed approximately 300–450 kg. The areas extending from tuber coxae to 5th intercostal space (ICS) and from dorsal midline to linea alba on left side for spleen and on right side for liver were shaved, respectively. Animals were secured in standing position in a cattle crate without any chemical restraint. The organs were examined with a 3.5 MHz curvilinear transducer. The distances between the dorsal midline of the body and the dorsal as well as the ventral ultrasonographic margins of the spleen and the liver were recorded with a measuring tape from the left 12th to 7th ICSs and the right 12th to 10th ICSs, respectively.

## 3. Results

### 3.1. Water Bath Study

**Figure 1:**
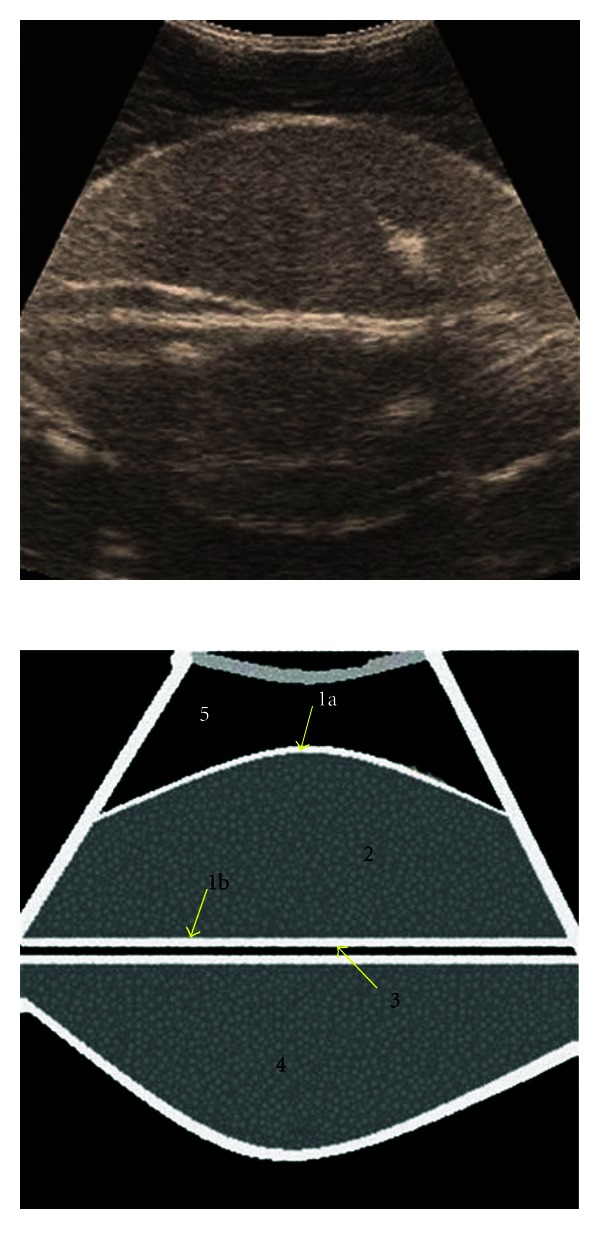
Ultrasonographic appearance of the spleen in transverse section in a water bath. 1a: parietal surface of splenic capsule, 1b: visceral surface of splenic capsule, 2: splenic parenchyma, 3: base of the plastic container, 4: mirror-image artifact, 5: water.

**Figure 2:**
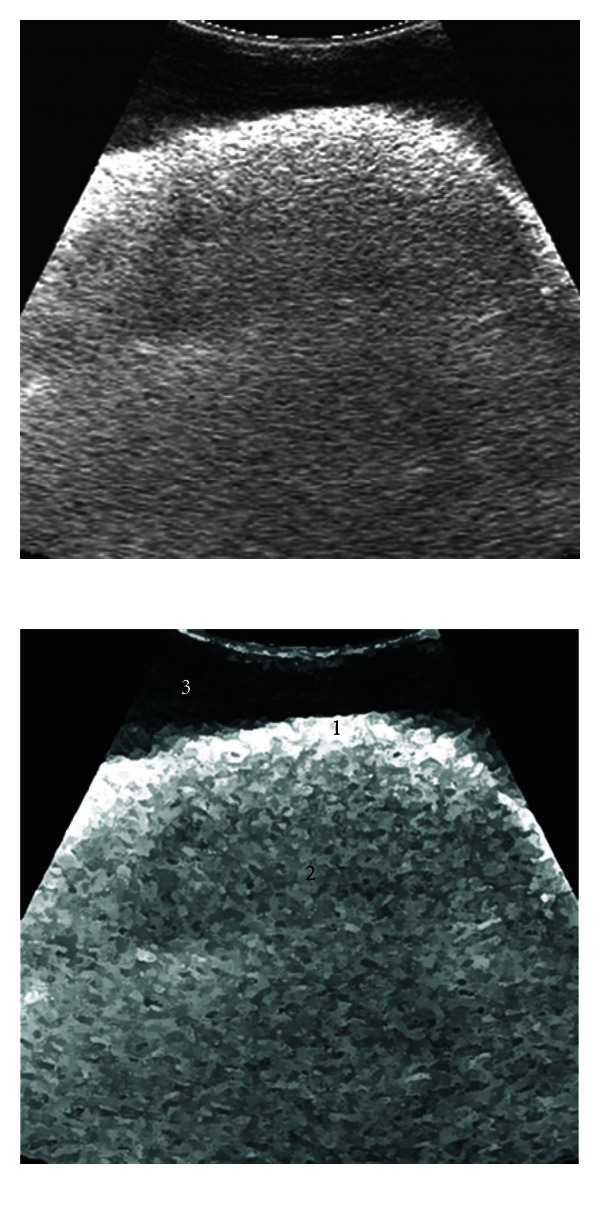
Ultrasonographic appearance of the liver in a water bath. 1: parietal surface of the liver, 2: liver parenchyma, 3: water. Notice the acoustic enhancement induced increase in echogenicity of the liver surface adjacent to the transducer.

The splenic parenchyma was seen consisting of isoechogenic granular pattern with an enveloping thick echogenic capsule (Figure 1). The splenic vessels were invisible. The mirror-image artifacts were clearly observed. The liver parenchyma was of coarse granular isoechogenicity, but the liver surface adjacent to the transducer appeared hyperechogenic (Figure 2). The liver capsule could not be identified with certainty. The hepatic vessels including the portal vein and the caudal vena cava were invisible; however, the vena caval sulcus was seen as triangular in cross-section. The gallbladder was seen just like a pear-shaped anechogenic structure with a hyperechogenic wall; however, the continuation of the cystic duct and the extrahepatic ducts could not be visualized. On a comparative basis, the echogenicity of the splenic parenchyma was greater than that of the hepatic parenchyma.

### 3.2. Ultrasonography of the Spleen and Liver in Healthy Cows

**Figure 3:**
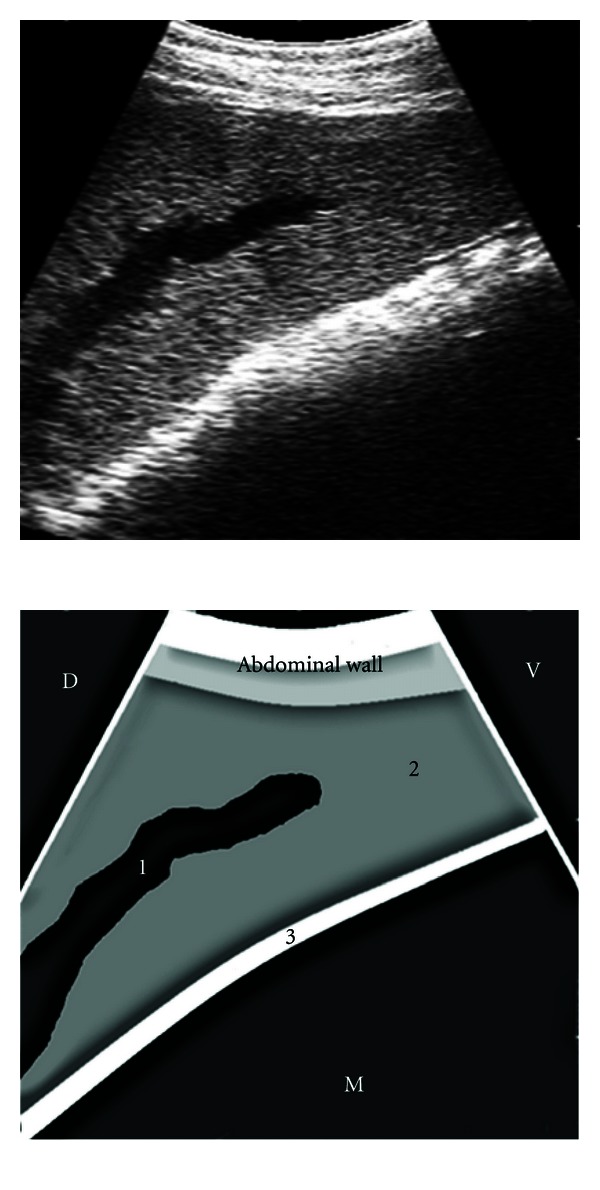
Ultrasonogram of the spleen obtained from the left 12th ICS by placing the transducer parallel to the ribs. 1: splenic vessel in longitudinal section, 2: splenic parenchyma, 3: wall of rumen, ICS: intercostal space, D: dorsal, V: ventral, M: medial.

**Figure 4:**
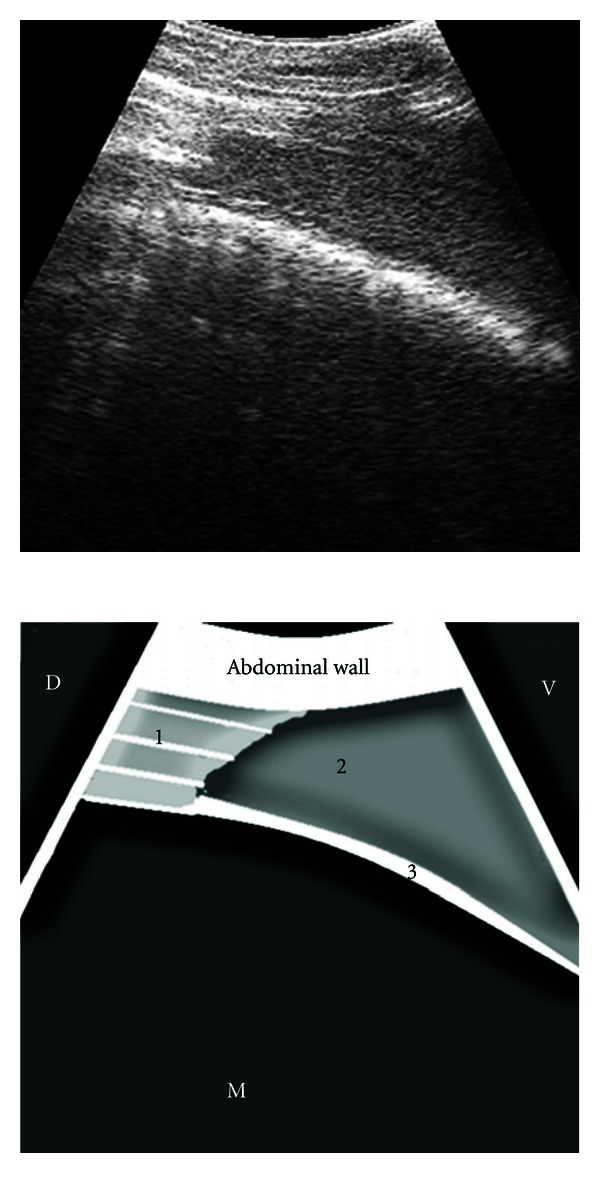
Ultrasonogram of the spleen, reticulum, and the left lung obtained from the left 5th ICS recorded during the reticular contraction, by placing the transducer parallel to the ribs. 1: ventral portion of lung depicting internal reverberation artifact, 2: spleen, 3: wall of reticulum, ICS: intercostal space, D: dorsal, V: ventral, M: medial.

The images of the splenic parenchyma obtained through the intercostal spaces were of homogeneous echogenicity and hyperechogenic with respect to the liver in the same cow. The splenic vessels running through the parenchyma were observed as branching anechogenic bands in the longitudinal plane or as anechogenic oval to circular structures in the transverse plane (Figure 3). The splenic capsule was seen as a thick echogenic line. The dorsal end of the spleen in the cranial intercostal spaces could be clearly seen at the peak of inspiration when the lungs retracted to uncover the parietal surface of the spleen. The ventral end of the spleen, portion of the left lung, and the reticular wall could be scanned in the left 7th to 6th ICS in all the cows, and in the left 5th ICS in 5 cows (Figure 4), by placing the transducer parallel to the ribs. The distance (mean ± SE) between the dorsal splenic margin and the midline was shortest in the 12th ICS (16.13 ± 0.83 cm) and greatest in the 7th ICS (51.88 ± 1.1 cm). Similarly, the distance between the ventral splenic margin and the midline was also shortest in the 12th ICS (24.13 ± 0.99 cm) and greatest in the 7th ICS (69.13 ± 2.5 cm). The mean dorsoventral extent of the spleen ranged from 8.0 cm in the 12th ICS to 18.5 cm in the 8th ICS (Table 1).

The liver was imaged from just behind the 12th to 6th ICSs in all the cows easily. The hepatic parenchyma was of homogeneous echogenicity interspersed with arborizing anechogenic bands of the hepatic vessels. Intermittent tiny hyperechogenic spots were also observed throughout the hepatic parenchyma. In general, there was slight variation in hepatic echotexture on an individual basis; however, when compared with the spleen, the liver was always of lesser echogenicity. The distances (mean ± SE) between the dorsal liver margin and the dorsal midline were 15.7 ± 1.36 cm in the 12th ICS, 23.0 ± 2.08 cm in the 11th ICS and 32.0 ± 2.15 cm in the 10th ICS. Similarly, the distance between the ventral liver margin and the dorsal midline was shortest in the 12th ICS, that is, 39.9 ± 2.08 cm and increased cranially, that is, 48.6 ± 3.13 cm in the 11th ICS and 49.3 ± 2.18 cm in the 10th ICS. The mean dorsoventral extent of the liver ranged from 17.30 to 25.60 cm, it was greatest in the 11th ICS (25.6 cm) and decreased cranially—17.30 cm in the 10th ICS and caudally—24.2 cm in the 12th ICS (Table 2).

**Figure 5:**
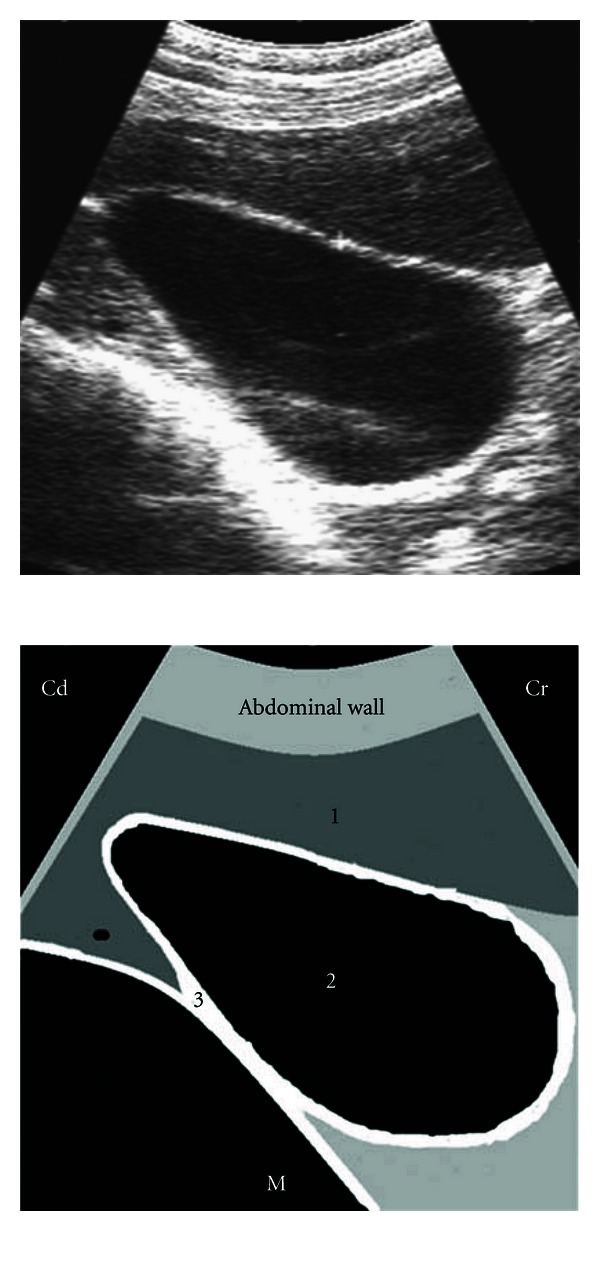
Ultrasonogram of the gallbladder and liver imaged from the ventral paracostal region of the 10th to 11th ICS by placing the transducer parallel to the longitudinal axis of the cow. 1: liver, 2: gallbladder, 3: omasum, ICS: intercostal space, Cr: cranial, Cd: caudal, M: medial.

**Figure 6:**
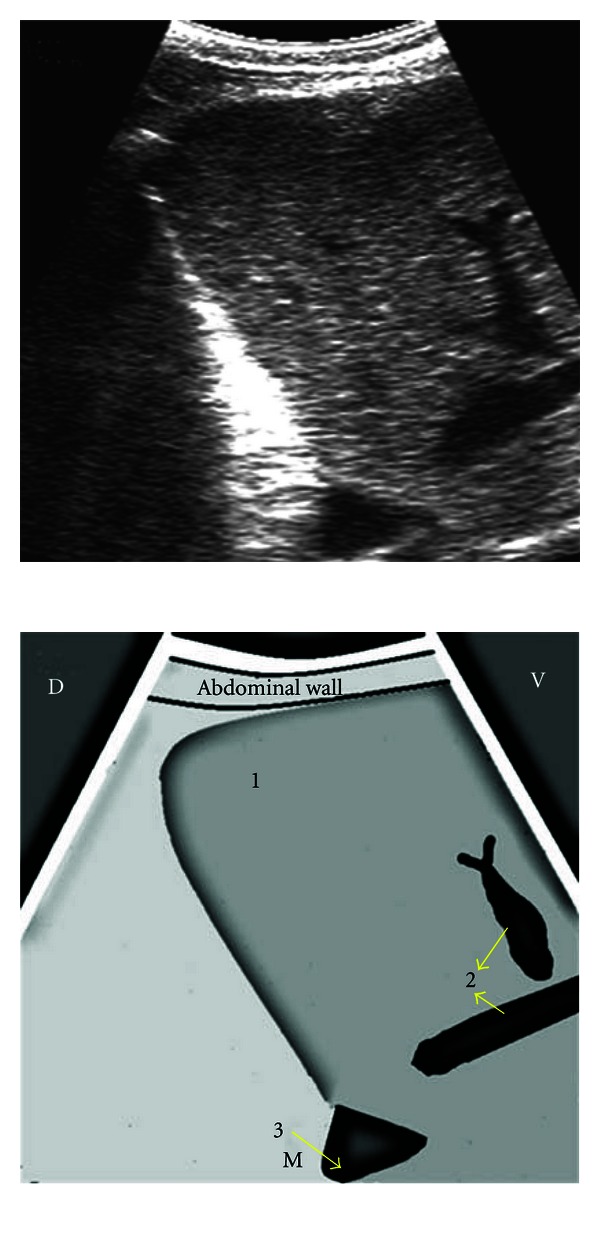
Ultrasonogram of the caudal vena cava obtained from the right 12th ICS by placing the transducer parallel to the ribs. 1: hepatic parenchyma, 2: hepatic veins, 3: caudal vena cava, ICS: intercostal space, D: dorsal, V: ventral, M: medial.

**Figure 7:**
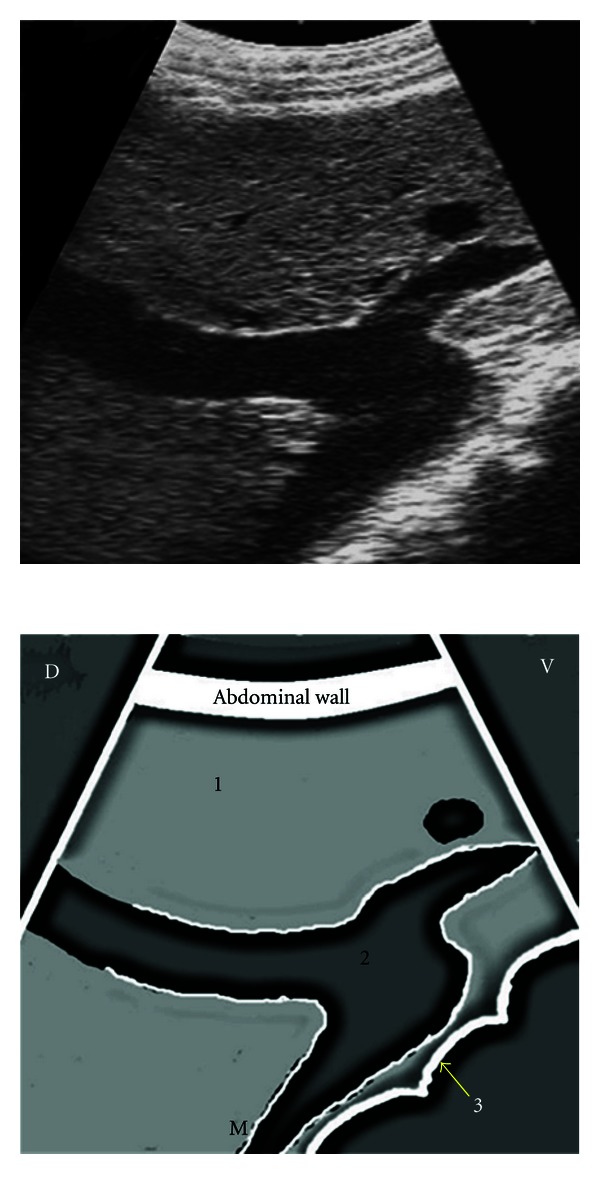
Ultrasonogram of the portal vein depicting its characteristic branching pattern obtained from the 11th ICS by placing the transducer parallel to the ribs. 1: hepatic parenchyma, 2: star-shaped branching of the portal vein, 3: wall of the colon, ICS: intercostal space, D: dorsal, V: ventral, M: medial.

The gallbladder was imaged as a tear-drop- or pear-shaped anechogenic structure with a hyperechogenic wall. The location of the gallbladder varied from the 12th to 9th ICS. It was most consistently observed in the 11th ICS (in 7 cows) at a mean distance of 46.3 cm from the dorsal midline. In 3 cows, the gallbladder was also seen ventral to the costal arch (Figure 5). The size of the gallbladder also varied widely as it was seen in 3 intercostal spaces (9th–11th ICS) in 4 cows, in 2 intercostal spaces (10th-11th ICS) in 5 cows, and limited to the 11th ICS in just 1 cow. The anechogenic cystic duct arising at the apex the gallbladder could be seen in only 1 cow. The mean wall thickness of the gallbladder was 2.5 mm. Artifacts resembling biliary suspensions were occasionally seen in the gallbladder; however, these were differentiated by thorough fanning of the ultrasound beam. Bile ducts could not be distinguished in the hepatic parenchyma in any of the cows. However, arc-shaped hyperechogenic mineralized structures, indicative of the bile ducts, with a marked distal shadow were observed in the liver parenchyma of 4 aged cows. 2 major vessels, namely, the caudal vena cava (CVC) and the portal vein (PV), were observed in all the cows. The CVC was imaged as a triangular anechogenic structure on the dorsal border of the liver in the last 2 intercostal spaces in all the cows (Figure 6). Cranially in the 10th ICS, it could be seen only in 3 cows as the overlying lungs hindered the imaging in rest of the cows. The diameters of the CVC in the 12th and the 11th ICS were 2.96 ± 0.14 cm and 3.05 ± 0.22 cm, respectively. The PV was imaged characteristically as a star-shaped anechogenic structure with a hyperechogenic wall, ventral to the CVC, in the last 2 intercostal spaces in all the cows and in the 10th ICS in only 8 cows (Figure 7). The diameters of the PV in the 12th, 11th, and 10th ICS were 3.11 ± 0.16, 2.87 ± 0.12, and 2.38 ± 0.12 cm, respectively.

## 4. Discussion

Water bath study provided a good learning experience for the comparative analysis of echotexture of the spleen and the liver. The echotexture of the splenic and hepatic parenchyma varied in the water bath study and in the live animals. The noticeable difference was the invisibility of the splenic and hepatic vessels in the water bath study. It was probably due to clotting of the blood in the vessels which had altered the echotexture in the water bath specimens. In contrast, the functional vessels in the live animals were easily identifiable due to anechogenicity of the circulating blood relative to the surrounding parenchyma. Further, replicate mirror-image artifacts were also recorded due to the reflection of the ultrasound beam at the water-plastic container base interface. Mirror-image artifacts are produced when an object is located in front of a highly reflective surface at which near total reflection takes place. Part of the energy is reflected back toward the reflector, and some of the energy is transmitted toward the transducer. This process is repeated so that multiple echoes separated in time are generated from the same object. The separation is equivalent to the distance between the object and the reflector [4].

Generally, the splenic and the hepatic parenchyma were granular and isoechogenic. It has been reported that the ultrasonographic appearances of the normal spleen and the liver range from a slightly coarsened appearance to a quite fine-grained texture varying from individual to individual [5]. In order to mitigate the apparent textural changes in the parenchyma, the confounding effects of the individual variation, intervening media or tissues, and the machine settings should be kept in mind [6]. When compared with the spleen, the liver should have a reduced echogenicity and a diffuse, slightly coarse-grained texture [5]. In the present study, though there were some individual differences in the echotexture of the liver parenchyma, yet it was always slightly hypoechogenic compared to the spleen. The hyperechogenicity of the liver surface adjacent to the transducer in the water bath study could be attributed to the acoustic enhancement caused by the water. Distal acoustic enhancement is caused by augmentation of the amplitude of the echoes distally to a structure with a low attenuation, more often fluid [6].

Braun and Götz [7] reported that the reticulum and the ventral part of the spleen could usually be imaged from the 6th and the 7th ICS on the left side of the cow. The spleen was visualized at approximately the level of the olecranon as further dorsally it could not be imaged due to the overlying lung. The differences regarding the imaging of the reticulum and the spleen in the left 5th ICS in our study may be attributed to the breed-based variation in the topographic anatomy. This has clinical significance since imaging of the spleen and the reticulum in the 5th ICS may lead to false positive diagnosis in the bovines suspected of diaphragmatic herniation [8].

Braun and Sicher [1] reported imaging of the spleen in 7th to 12th ICS. The long axis of spleen was found to be oblique and running caudodorsal to cranioventral. The distance from the dorsal margin of the spleen to the midline of the back was greatest in the 7th ICS (60.9 ± 6.81 cm) and smallest in the 12th ICS (12.7 ± 2.85 cm). The extent of the spleen was greatest in the 8th ICS (24.9 ± 10.77 cm) and smallest in the 12th ICS (9.5 ± 5.38 cm). Similarly, Braun [3] reported that the size of the liver appeared about the same in the 12th and the 11th ICS (24.9 ± 3.89 and 26.9 ± 2.94 cm, resp.) but was notably smaller in the 10th ICS (20.7 ± 3.11 cm). Measurements of these variables were not significantly different among three heavy breeds of cattle (Swiss Braunvieh, Simmental, and Holstein) or among those of differing ages (2.5–11.5 years), but differences were noted for various body weights, milk production, and stages of gestation; there was a tendency for heavier cows to have thicker and larger liver than did the lighter cows and the cows with lower milk production [9]. The dimensions of the splenic and hepatic sizes obtained in the current study were on a lower side in comparison to the aforementioned studies. We believe that the breed- and bodyweight-based differences played an important role in the determination of the sizes of the spleen and the liver.

The location of the gallbladder varied from the 12th to 9th ICS, and its relative distance from dorsal midline also varied. Further, apart from the intercostal spaces, it was also imaged ventral to the costal arch in 3 animals. However, these findings are somewhat at variation to those reported by others [3, 9]. Biliary suspension artifacts observed in our study resulted from the ultrasonographic interaction with the tissues. Side-lobe artifact may mimic material within the gallbladder which can be corrected by decreasing the power or the gain [6].

The mean diameters of the CVC and the PV obtained in the current study were on a lower side as reported previously [3, 9]. The typical star-shaped PV and its branches were characterized by a thin hyperechogenic wall and anechogenic lumen. The CVC was triangular in shape and was located more dorsal and medial than the portal vein. The walls of the CVC and hepatic veins were not distinct. These findings were in consonant with the previous studies [3, 9].

The ultrasonographic appearance of the CVC is a substantial aid in diagnosing congestion in the systemic circulation. The cross-sectional image of the CVC assumes more oval to circular shape instead of triangular in the patients with impaired venous return [10]. In addition, the ultrasonographically determined dorsoventral extents of the liver increase in cases of the end-stage congestive heart failure which can be properly ascertained only after developing a normal baseline data in healthy animals [10]. The most common cause of the bile duct calcification is the chronic fascioliasis. Calcified bile ducts result in the discrete sonographic changes in the liver parenchyma. This could have been the possible reason for the distal acoustic shadow emanating from the liver parenchyma of the previously mentioned 4 aged cows. In cross-section, the calcified bile ducts appear as ring-like, and in longitudinal section they appear as tube-like hyperechogenic structures [11]. Acoustic shadowing appears as an anechoic area distal to a structure that strongly attenuates the ultrasound, such as bone or any mineralized or dense material [6].

Normalization of these parameters based on cow size would be a useful way to compare studies and develop criteria that could be applied to cows of different breeds and body weights. Therefore, making an assessment of the dorsoventral extents of the diseased spleen/liver in order to arrive at a diagnosis without considering the aforementioned variables should be evaluated with caution. It was concluded that a systematic ultrasonography formed a basis for a reliable noninvasive determination of positions and sizes of the normal spleen and liver and their vessels in the bovines.

## Figures and Tables

**Table 1:** The distances of the splenic dorsal and ventral margins from the dorsal midline in 10 cows, mean ± SE.

Measurement (cm)	12th ICS	11th ICS	10th ICS	9th ICS	8th ICS	7th ICS
Dorsal margin	16.13 ± 0.83	20.5 ± 1.6	30.5 ± 1.42	38.34 ± 1.47	44.34 ± 1.24	51.88 ± 1.1
Ventral margin	24.13 ± 0.99	34.13 ± 1.48	44.5 ± 1.22	53.63 ± 1.69	62.88 ± 2.76	69.13 ± 2.5
Mean dorsoventral extent	8.0	13.63	14.0	15.25	18.5	17.25

**Table 2:** The distances of the dorsal and ventral margins of the liver from the dorsal midline in 10 cows, mean ± SE.

Measurement (cm)	12th ICS	11th ICS	10th ICS
Dorsal margin	15.7 ± 1.36	23.0 ± 2.08	32.0 ± 2.15
Ventral margin	39.9 ± 2.08	48.6 ± 3.13	49.3 ± 2.18
Mean dorsoventral extent	24.2	25.6	17.3
